# Extracellular Vesicle IL5RA and BCMA in Serum Enable Non-Invasive Risk Stratification of Multiple Myeloma

**DOI:** 10.3390/cancers18071116

**Published:** 2026-03-30

**Authors:** Yuko Shirouchi, Hiroki Shinchi, Yoshimi Haga, Yuko Mishima, Sayuri Minowa, Tomoko Takayama, Shunji Takahashi, Dai Maruyama, Koji Ueda

**Affiliations:** 1 Division of Analytical Biochemistry, Cancer Precision Medicine Center, Japanese Foundation for Cancer Research, 3-8-31, Ariake, Koto, Tokyo 135-8550, Japan; yuko.shirouchi@jfcr.or.jp (Y.S.); hiroki.shinchi@jfcr.or.jp (H.S.); yoshimi.haga@jfcr.or.jp (Y.H.); 2 Department of Hematology Oncology, Cancer Institute Hospital, Japanese Foundation for Cancer Research, 3-8-31, Ariake, Koto, Tokyo 135-8550, Japan; yuko.mishima@jfcr.or.jp (Y.M.); dai.maruyama@jfcr.or.jp (D.M.); 3 Cancer Bioscience/Oncotherapeutic Medicine, Tohoku University School of Medicine, 2-1 Seiryo-machi, Aoba-ku, Sendai 980-8575, Miyagi, Japan; s.takahashi-chemotherapy@jfcr.or.jp; 4 The Cancer Chemotherapy Center, Division of Clinical Research, Japanese Foundation for Cancer Research, 3-8-31, Ariake, Koto, Tokyo 135-8550, Japan; sminowa@jfcr.or.jp (S.M.); tomoko.takayama@jfcr.or.jp (T.T.); 5 Department of Medical Oncology, Cancer Institute Hospital, Japanese Foundation for Cancer Research, 3-8-31, Ariake, Koto, Tokyo 135-8550, Japan

**Keywords:** multiple myeloma, extracellular vesicles, proteomics, biomarker

## Abstract

**Background/Objectives**: Multiple myeloma (MM) is an incurable plasma cell neoplasm in which diagnosis and prognostication rely on invasive bone marrow examinations that may not capture biological heterogeneity across different disease sites. There is a clinical need for non-invasive biomarkers that can accurately predict treatment outcomes. **Methods**: We performed a global proteomic profiling of bone marrow-derived extracellular vesicles (EVs) from nine MM patients and ten controls. A total of 8839 proteins were identified, of which 14 met predefined selection criteria. These candidates were quantified in serum-derived EVs using targeted proteomic analysis. Prognostic relevance of selected proteins was evaluated in newly diagnosed MM (NDMM) patients treated with daratumumab-containing frontline regimens (*n* = 26) and healthy individuals (*n* = 60). Progression-free survival (PFS) was analyzed using univariable and multivariable models. **Results**: IL5RA (*p* = 0.003) and BCMA (*p* < 0.001) were significantly elevated in serum EVs from MM patients compared with controls. Higher serum EV-IL5RA and EV-BCMA were associated with a trend toward shorter PFS. Combined assessment of these biomarkers enabled clear stratification of MM patients into three prognostic groups, including a cohort with markedly inferior outcomes, with a 20-month PFS of 0 (*p* = 0.001). In multivariable analysis, the combined serum EV-IL5RA and EV-BCMA signature suggests an independent prognostic potential (HR = 38.49 [95% CI, 1.51–47.79], *p* = 0.015). **Conclusions**: Serum EV-IL5RA and EV-BCMA are novel non-invasive biomarkers, measurable through routine blood testing, with strong potential to improve risk stratification in NDMM patients in the era of daratumumab-based frontline therapy.

## Simple Summary

Diagnosis and prognostication of multiple myeloma currently rely on bone marrow examination, which is invasive and may not fully reflect biological heterogeneity across different disease sites. This research was conducted to develop less invasive blood-based biomarkers that better capture disease biology and predict patient outcomes. We first performed global proteomics of bone marrow-derived extracellular vesicles and identified 8839 proteins, of which 14 met predefined selection criteria. These candidates were subsequently validated in serum-derived extracellular vesicles. Two proteins, IL5RA and BCMA, were elevated in myeloma patients compared with healthy individuals. Furthermore, their combined measurement separated newly diagnosed myeloma patients receiving daratumumab-containing frontline therapy into distinct risk groups, including one with very poor outcomes. These findings support IL5RA and BCMA in the serum extracellular vesicles as novel non-invasive biomarkers, with potential to improve risk stratification in multiple myeloma in the modern treatment era.

## 1. Introduction

Multiple myeloma (MM) is a plasma cell neoplasm, with an incidence of approximately six cases per 100,000 individuals and accounting for an estimated 4500 deaths annually in Japan [1]. The introduction of novel therapeutic agents, including immunomodulatory drugs, proteasome inhibitors, and anti-CD38 monoclonal antibodies, has significantly improved the prognosis of newly diagnosed MM (NDMM) patients in recent years [2,3,4,5]. In particular, the incorporation of the anti-CD38 monoclonal antibody, daratumumab, into frontline regimens has led to marked improvements in both progression-free survival (PFS) and overall survival (OS) [6,7]. However, MM remains incurable, and most patients experience multiple relapses over the course of their disease.

Current diagnostic and prognostic assessments of MM rely heavily on bone marrow evaluation [8,9,10]; however, such procedures are not only invasive but may also fail to capture biological heterogeneity across different disease sites [11]. Therefore, there is a need for less invasive approaches to obtain diagnostic and prognostic information. Extracellular vesicles (EVs) are lipid bilayer-enclosed particles secreted by cells, including malignant ones. They encapsulate diverse biomolecules such as proteins, nucleic acids (e.g., RNA, DNA), and lipids, and play essential roles in intercellular communication [12,13]. EVs have thus emerged as promising sources for liquid biopsy in oncology [14,15].

In this study, we conducted a comprehensive proteomic analysis, first using global proteomics of bone marrow-derived EVs, followed by targeted proteomic analysis of serum-derived EVs, to identify diagnostic and prognostic biomarkers detectable through routine, non-invasive blood testing in patients with NDMM treated with daratumumab-containing frontline regimens.

## 2. Materials and Methods

### 2.1. Patients and Sample Collection

Patients newly diagnosed with MM at Cancer Institute Hospital, Japanese Foundation for Cancer Research, between January 2022 and August 2024 were prospectively enrolled in this study. The diagnosis was made according to International Myeloma Working Group criteria [8,9]. Bone marrow (EV proteomics, *n* = 9; RT-qPCR, *n* = 21) and serum samples (*n* = 26) were collected from NDMM patients at the time of diagnosis. For serum samples, the median time from sample collection to initiation of daratumumab-containing therapy was 0 days (interquartile range, 0–1.75), indicating that the majority of samples were obtained on the day of treatment initiation. Control samples for each analysis were as follows: bone marrow samples from lymphoma patients without bone marrow involvement (*n* = 10) and serum samples from healthy individuals (*n* = 60). The detailed information for each sample is provided in Table S1. Clinical and pathological information was obtained from electronic medical records and institutional databases. All participants provided written informed consent prior to enrolment. This study was approved by the institutional review board of the Japanese Foundation for Cancer Research (2015–1157) and was conducted in accordance with the Declaration of Helsinki.

### 2.2. Bone Marrow Sample Preparation

Bone marrow aspirate (2 mL) was collected and centrifuged at 3000 rpm for 15 min at room temperature. The supernatant was obtained and stored at −80 °C until further analysis. CD138-positive plasma cells were isolated from bone marrow aspirates using magnetic-activated cell sorting (MACS) with an autoMACS Pro Separator (Miltenyi Biotec, Bergisch Gladbach, Germany), according to the manufacturer’s protocol, using CD138 microbeads (#130-105-961 and #130-051-301, Miltenyi Biotec).

### 2.3. EV Isolation

EVs were isolated from bone marrow supernatants and serum using a phosphatidylserine-binding affinity method with MagCapture™ Exosome Isolation Kit PS Ver. 2 (FUJIFILM Wako, Chuo-Ku, Japan), according to the manufacturer’s instructions. Briefly, 200 µL of thawed bone marrow supernatant or serum was diluted 1:5 with phosphate-buffered saline (PBS). The diluted samples were incubated with magnetic beads at room temperature for 60 min. After incubation, the beads were washed three times on a magnetic stand. For subsequent LC-MS and Western blot analysis, EVs were eluted in 30 μL of Laemmli’s sample buffer (25 mM Tris, 1% SDS, 4% Glycerol, 2% 2-Mercaptoethanol, 0.01% Bromophenol Blue). For protein quantification, EVs were eluted with 30 µL of phase transfer surfactants (PTS) buffer (12 mM sodium deoxycholate and 12 mM N-lauroyl sarcosinate in PBS) and protein concentrations were measured using the BCA Micro kit (Thermo Fisher Scientific, Waltham, MA, USA).

### 2.4. Western Blot

EVs were lysed with Laemmli’s sample buffer for protein denaturation, and the proteins were separated using 10% Bolt gels, followed by transfer to polyvinylidene difluoride (PVDF) membranes. Membranes were blocked with 4% Block ACE (#UKB80, Megmilk Snow Brand, Tokyo, Japan) and incubated overnight with the following primary antibodies: anti-CD9 and anti-CD63 (mouse monoclonals, Cosmo Bio, Tokyo, Japan, 1:2000 dilution), and anti-haptoglobin (rabbit polyclonal, Abcam, Cambridge, UK, 1:1000 dilution). After washing, membranes were treated with HRP-conjugated secondary antibodies (anti-mouse IgG or anti-rabbit IgG, Cytiva, Marlborough, MA, USA, 1:10,000 dilution). Protein signals were visualized using Western Lightning ECL Pro (#NEL121001EA) (Revvity, Waltham, MA, USA) and chemiluminescence images were obtained using the ChemiDoc™ Touch Imaging System (Bio-Rad, Hercules, CA, USA).

### 2.5. Nanoparticle Tracking Analysis (NTA)

EVs were diluted 1:50 in PBS and subjected to NTA using a ZetaView system (Particle Metrix, Inning am Ammersee, Germany) equipped with a 488 nm laser. Particle size distribution and concentration were analyzed with ZetaView software version 8.05.16 SP3.

### 2.6. Protein Digestion and LC-MS Analysis

Proteins were recued using 10 mM TCEP at 100 °C for 10 min, followed by alkylation with 50 mM iodoacetamide at room temperature for 45 min. Proteins were subsequently digested with Trypsin/Lys-C Mix (Promega, Madison, WI, USA) on S-Trap columns (ProtiFi, Fairport, NY, USA) at 47 °C for 2 h.

The resulting peptides were subjected to LC-MS analysis using Orbitrap Fusion Lumos mass spectrometer (Thermo Scientific) coupled to UltiMate 3000 RSLC nano-flow HPLC (Thermo Scientific). Peptides were enriched with μ-Precolumn (0.3 mm i.d. × 5 mm, 5 μm, Thermo Scientific) and separated on AURORA column (0.075 mm i.d. × 250 mm, 1.6 μm, Ion Opticks Pty Ltd., Collingwood, Australia). Peptide separation was performed using a two-step gradient: 2–30% acetonitrile for 110 min, followed by 30–95% acetonitrile for 5 min in the presence of 0.1% formic acid. The compensation voltages for gas-phase fractionation by FAIMS Pro (Thermo Scientific) were set at −40, −60, and −80 V. The analytical parameters of Orbitrap Fusion Lumos were set as follows: Resolution of full scans = 50,000, Scan range (*m*/*z*) = 350–1500, Maximum injection time of full scans = 50 msec, AGC target of full scans = 4 × 10^5^, Dynamic exclusion duration = 30 s, Cycle time of data dependent MS/MS acquisition = 2 s, Activation type = HCD, Detector of MS/MS = Ion trap, Maximum injection time of MS/MS = 35 msec, AGC target of MS/MS = 1 × 10^4^. The MS/MS spectra were searched against the Homo sapiens protein sequence database in UniProt using Proteome Discoverer 3.1 software (Thermo Scientific), with peptide identification filtered at a false discovery rate <1%. Label-free relative quantification analysis for proteins was performed with the default parameters of the Minora Feature Detector node, Feature Mapper node, and Precursor Ions Quantifier node in Proteome Discoverer 3.1 software.

### 2.7. Multiple Reaction Monitoring (MRM)

Targeted MRM analysis was performed using an LCMS-8060 triple quadrupole mass spectrometer (Shimadzu, Kyoto, Japan) coupled to an UltiMate 3000 RSLC nano system. Peptides were enriched with μ-Precolumn (0.3 mm i.d. × 5 mm, 5 μm, Thermo Scientific) and separated on AURORA ELITE column (0.075 mm i.d. × 150 mm, 1.7 μm, Ion Opticks Pty Ltd., Australia). Peptide separation was performed using a two-step linear gradient (2–30% acetonitrile for 15 min, 30–95% for 2 min, in 0.1% formic acid), at a flow rate of 300 nL/min. The LCMS-8060 instrument was operated under the following conditions: nebulizer gas (0.5 L/min), drying and heating gas (10.0 L/min each), ESI interface (2.0 kV), interface temperature (300 °C), DL temperature (200 °C), heat block temperature (200 °C), and CID gas pressure (300 kPa). The optimized MRM transitions used in this study are listed in Table S6. Stable isotope-labeled peptides synthesized by Scrum Inc. (Tokyo, Japan) were added to the samples as internal standards (Table S3). Peptide quantification was determined from the peak area ratio of endogenous peptides to their corresponding isotope-labeled standards, using LabSolutions v5.97 (Shimadzu, Kyoto, Japan).

### 2.8. RNA Extraction and RT-qPCR

Total RNA was extracted from CD138-positive cells using the RNeasy Mini or Micro Kit (Qiagen, Venlo, The Netherlands). cDNA was generated using SuperScript III Reverse Transcriptase (Invitrogen, Carlsbad, CA, USA, #18090050). A cDNA panel of 48 normal human tissues was purchased from OriGene (Rockville, MD, USA) (HMRT104). RNA concentrations were measured using a NanoDrop Lite spectrophotometer (Thermo Fisher Scientific). RT-qPCR was performed with TaqMan™ Fast Advanced Master Mix (Thermo Fisher Scientific, #4444556) on a QuantStudio 3 system. Input cDNA amounts were 100 pg for BCMA and 2–5 ng for IL5RA. TaqMan assays included BCMA (Hs00171292_m1), IL5RA (Hs01064360_m1), and GAPDH (Hs02786624_g1). Relative gene expression levels were calculated after normalization to GAPDH using the 2^−ΔCT^ method.

### 2.9. Serum Soluble BCMA ELISA

Serum samples were diluted 1:100 and subjected to ELISA analysis using the Human BCMA/TNFRSF17 DuoSet ELISA Kit (#DY193E, R&D Systems, Minneapolis, MN, USA), according to the manufacturer’s instructions. Absorbance values were recorded at 450 nm using a SUNRISE microplate reader (TECAN, Männedorf, Switzerland) with XFLUOR4 software v4.51.

### 2.10. Statistical Analysis

For bone marrow EV proteomic analysis, two-tailed Welch’s *t*-tests were used to compare the MM and control groups. Candidate biomarkers were selected based on the following criteria: (1) For MM-exclusive proteins: ≥2 unique peptides and ≥50% valid values. (2) For significantly upregulated proteins: *p* < 0.05, FC ≥ 10, ≥2 unique peptides, and ≥50% valid values.

For serum EV analysis, differences in peptide concentration between MM patients and healthy controls were determined using the Mann–Whitney U test. Spearman’s rank correlation analysis was used to evaluate associations between EV-derived biomarkers and clinical variables, as well as correlations between biomarker levels measured in bone marrow and serum samples. PFS was estimated using the Kaplan–Meier method, and differences between groups were assessed using the log-rank test. Multivariable analysis was conducted using Cox proportional hazards regression. Multicollinearity among variables included in the model was assessed using variance inflation factors (VIF). PFS was defined as the time from diagnosis to disease progression or death. Statistical analyses were performed using R software (v4.4.0) with the EZR package. Statistical significance was defined as *p*-value < 0.05.

## 3. Results

### 3.1. Isolation of EVs from Bone Marrow and Serum

**Figure 1:**
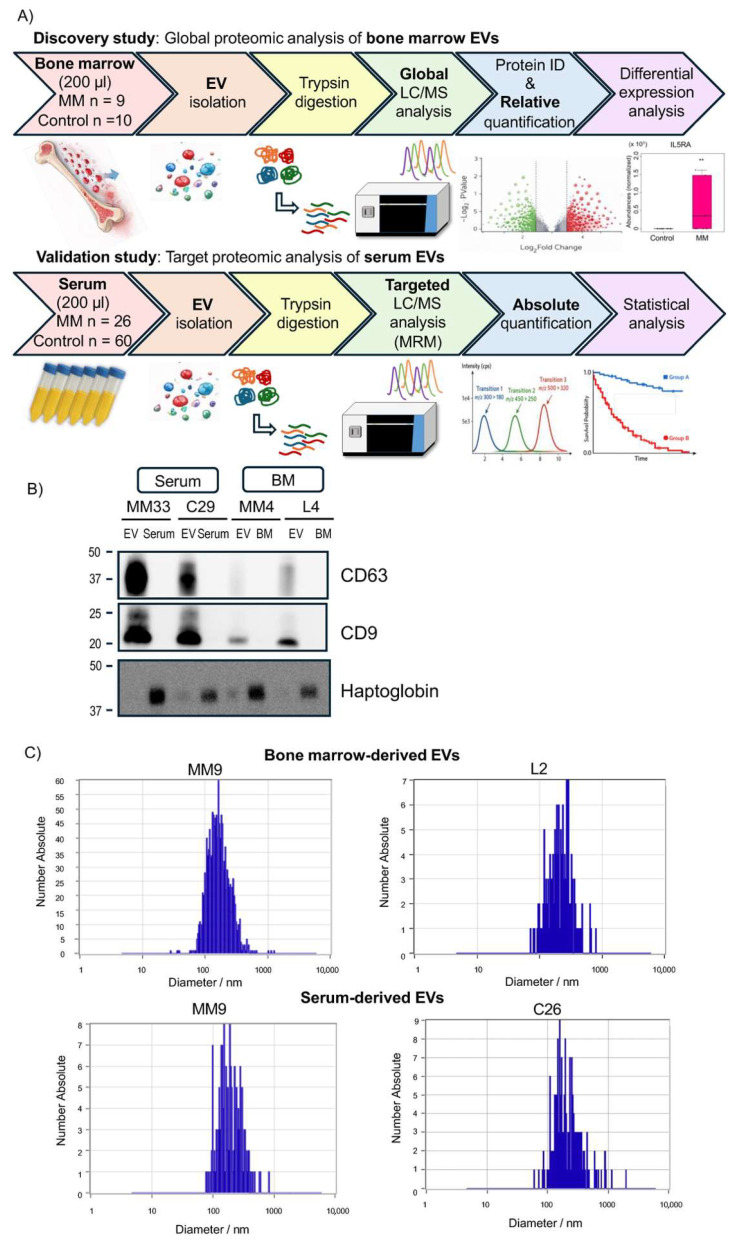
Isolation of EVs from bone marrow and serum. (**A**) Schematic overview of the experimental workflow for EV isolation from bone marrow and serum, followed by proteomic analysis. ** *p* < 0.01. (**B**) Western blot analysis of EV marker proteins CD9 and CD63 in isolated EVs. Non-EV free protein, Haptoglobin, was also blotted. Data are shown for serum-derived EVs from MM33 and C29, and bone marrow-derived EVs from MM9 and L2. (**C**) Representative nanoparticle tracking analysis (NTA) results for EVs. Data are shown for bone marrow-derived EVs from MM9 and L2, and serum-derived EVs from MM9 and C26.

A schematic overview of the proteomic analysis workflow is shown in Figure 1A. EVs were isolated using a TIM4 affinity-based method. Western blotting confirmed the enrichment of EV marker proteins, CD9 and CD63, in the EV samples compared to total bone marrow aspirate or serum (Figure 1B and Figure S1). Furthermore, nanoparticle tracking analysis (NTA) revealed that the median size distribution of EVs isolated from bone marrow and serum ranged from 150 to 200 nm (Figure 1C). These findings confirm the successful isolation of high-quality EVs from both bone marrow aspirate and serum, validating their suitability for downstream proteomic analyses.

### 3.2. Proteomic Characterization of Bone Marrow-Derived EVs

**Figure 2:**
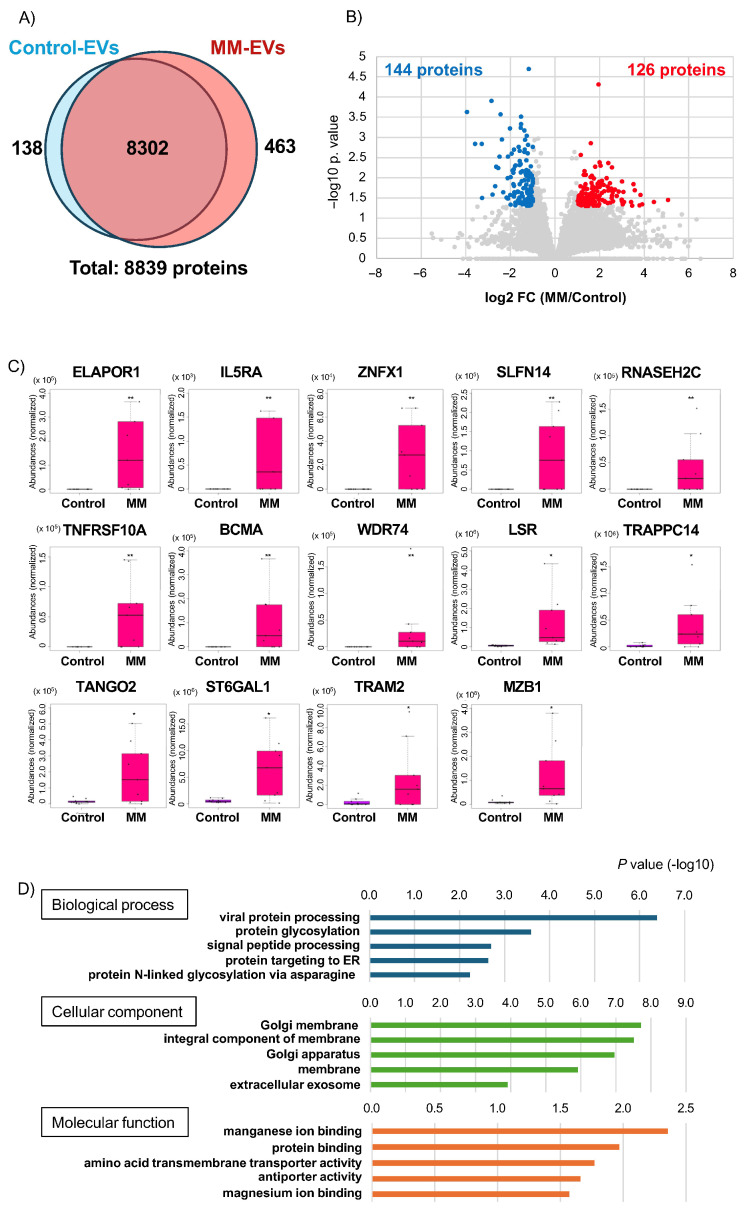
Label-free quantification of EVs isolated from bone marrow aspirates. (**A**) Venn diagram showing the number of proteins detected in bone marrow EVs from MM patients and controls by LC-MS analysis. (**B**) Volcano plot illustrating differentially expressed proteins in bone marrow EVs between MM patients and controls. Proteins significantly elevated (fold change ≥ 2, *p* < 0.05) in MM patients are highlighted in red, and proteins significantly decreased (fold change ≤ 0.5, *p* < 0.05) are shown in blue. Statistical significance was assessed using two-tailed Welch’s *t*-test. (**C**) Box plots showing normalized abundances of representative proteins that were either exclusively detected or significantly upregulated in bone marrow EVs of MM patients. Black dots represent individual data points. * *p* < 0.05, ** *p* < 0.01 by Welch’s *t*-test. (**D**) Gene ontology enrichment analysis (DAVID) of the 126 significantly upregulated proteins in bone marrow EVs from MM patients.

Bone marrow aspirate samples were collected at diagnosis from nine MM patients (Table S1A), and samples from ten lymphoma patients without bone marrow involvement served as controls (Table S2A). Label-free quantitative proteomic analysis of EVs from MM and control samples identified 8839 proteins (Figure 2A, Table S4). Comprehensive proteomic profiling of bone marrow supernatant-derived extracellular vesicles (EVs) revealed the presence of markers corresponding to multiple hematopoietic and stromal cell lineages. Specifically, we identified T-cell markers (CD3, CD4, CD5, CD8), B-cell markers (CD19, CD20, CD22, CD79a/b), NK-cell markers (CD56, NKG2D), monocyte/macrophage markers (CD11b, CD14, CD68, CD163), and neutrophil markers (CD33, MPO), as well as proteins associated with megakaryocytes/platelets (platelet glycoproteins, PF4) and the erythroid lineage (glycophorins, hemoglobin subunits). Additionally, we detected markers of bone marrow stromal cells (ENG, NT5E, THY1) and osteoblasts (ALPL, COL1A1) (Table S4).

Importantly, this diverse repertoire of EV-associated markers underscores the complexity of the bone marrow microenvironment as captured by whole-bone marrow EV profiling, which cannot be fully recapitulated by analyses limited to specific cell cultures. These findings indicate that bone marrow-derived EVs collectively carry components from a wide range of cells in the microenvironment, offering an integrated view of the bone marrow as it exists in situ.

Altogether, this dataset represents one of the first comprehensive EV proteomes characterizing the native bone marrow environment and provides a valuable foundation for future studies exploring the physiological and pathological roles of bone marrow-derived EVs.

### 3.3. Differential Expression Analysis of Bone Marrow-Derived EVs and Selection of Biomarker Candidate

Of the 8839 proteins identified, 463 were uniquely present in MM-derived EVs (Figure 2A and Table S5A), and 138 were exclusive to control-derived EVs (Figure 2A and Table S5B). Moreover, 126 proteins were significantly upregulated (fold change [FC] ≥ 2, *p* < 0.05), while 144 proteins were downregulated (FC ≤ 0.5, *p* < 0.05) in MM samples relative to controls (Figure 2B and Table S6A,B).

Gene ontology analysis of the 126 upregulated proteins revealed enrichment in biological processes related to “viral protein processing”, including glycosyltransferases (e.g., ST6GAL1, MGAT2) and glycosyl hydrolases (e.g., MAN2A1, MAN1B1), as well as signal peptide complex subunits (Figure 2D and Table S7A). These proteins are involved in the processing and secretion of glycoproteins, a hallmark of MM pathogenesis characterized by the excessive production of monoclonal immunoglobulins by malignant plasma cells. Many of the upregulated proteins were localized to the Golgi apparatus or cellular membranes, highlighting their functional roles in glycosylation and immunoglobulin trafficking (Figure 2D and Table S7B). Additionally, ion-binding activities required for enzymatic function were also overrepresented (Figure 2D and Table S7C).

From the 463 MM-specific proteins, we selected eight candidates that met the criteria of ≥2 unique peptides and ≥50% valid values (Table 1). From the 126 significantly upregulated proteins, six additional candidates met stricter criteria of ≥2 unique peptides, ≥50% valid values, and FC > 10 (Table 2). Altogether, 14 proteins were nominated as potential biomarker candidates (Figure 2C), including several previously associated with MM, such as TNFRSF17 (BCMA), interleukin-5 receptor alpha (IL5RA), and Marginal zone B and B1 cell-specific protein (MZB1). IL5RA and MZB1 have been implicated in MM cell proliferation [16,17,18]. MZB1 is involved in IgM assembly and secretion and is highly expressed in marginal zone B and B1 cells [19,20]. IL5RA, a receptor subunit for IL-5, plays roles in both eosinophil activation and B-cell maturation [21,22,23]. Another candidate, TNFRSF10A (TRAIL receptor 1), mediates apoptosis via TRAIL and has been explored in MM and other cancers [24,25]. Consequently, the 14 proteins, including those potentially related to the pathogenesis of MM, proceeded to further validation.

### 3.4. Identification of Serum EV Biomarkers via Targeted Proteomic Analysis

**Figure 3:**
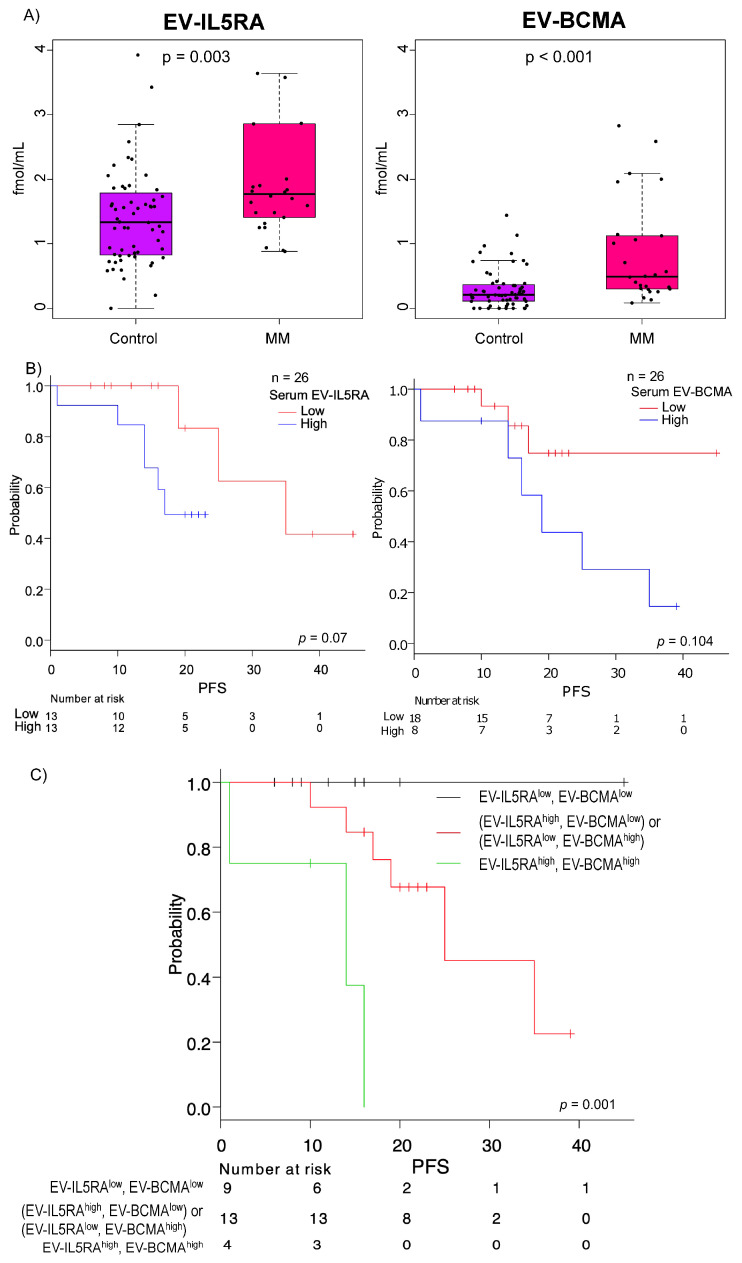
Targeted proteomic analysis of serum-derived EVs. (**A**) Box plots showing levels of serum EV-IL5RA and EV-BCMA in MM patients and healthy individuals. Black dots represent individual data points. *p*-values were calculated using the Mann–Whitney U test. (**B**) Kaplan–Meier curves for PFS of MM patients, stratified by baseline serum EV-IL5RA (left) and EV-BCMA (right) levels. Patients were categorized into high and low groups according to the cut-off values determined by ROC curve analysis. (**C**) Kaplan–Meier analysis of PFS based on combined stratification of serum EV-IL5RA and EV-BCMA levels. Patients were classified into three groups: EV-IL5RA^low^, EV-BCMA^low^, (EV-IL5RA^high^, EV-BCMA^low^) or (EV-IL5RA^low^, EV-BCMA^high^), and EV-IL5RA^high^, EV-BCMA^high^.

Given the invasiveness and occasional inadequacy of bone marrow sampling, we next evaluated whether the 14 biomarker candidates could be detected in serum EVs. Serum samples from 26 NDMM patients who were treated with daratumumab-containing regimens and from 60 healthy individuals were analyzed (Tables S1B and S2B). Absolute quantification was performed using multiple reaction monitoring (MRM) with stable isotope-labeled internal standards. Two proteins, IL5RA (FC = 1.33, *p* = 0.003) and BCMA (FC = 2.33, *p* < 0.001), were significantly elevated in serum-derived EVs of MM patients (Figure 3A). These results indicate that IL5RA and BCMA are not only detectable in serum-derived EVs but are also quantitatively elevated at diagnosis, supporting their potential utility as clinically applicable blood-based biomarkers for MM.

Furthermore, both serum EV-IL5RA and EV-BCMA showed a positive correlation trend with their levels in bone marrow EVs (Spearman’s correlation coefficient = 0.60 and 0.80, respectively). In addition, patients with high EV-IL5RA or EV-BCMA tended to have higher M-protein levels, and when combined, these biomarkers further stratified M-protein levels and reached statistical significance (*p* = 0.04), suggesting that these EV markers may reflect tumor burden (Figure S2).

### 3.5. Serum EV-IL5RA and EV-BCMA as an Independent Prognostic Biomarker

Receiver operating characteristic (ROC) curves were used to define prognostic cut-off values for serum EV-IL5RA and EV- BCMA (Figure S3A,B). With a median follow-up of 20 months, patients with elevated baseline serum EV-IL5RA (≥1.80 fmol/mL) or EV-BCMA (≥1.06 fmol/mL) exhibited a trend toward shorter PFS compared to those without elevation of these corresponding biomarkers, although the differences did not reach statistical significance (Figure 3B). Specifically, patients with high serum EV-IL5RA had a 20-month PFS of 49.4% (95% CI, 19.7–73.6) compared with 83.3% (95% CI, 27.3–97.5) in those with low serum EV-IL5RA (*p* = 0.07). Similarly, patients with high serum EV-BCMA had a 20-month PFS of 43.8% (95% CI, 10.1–74.2), whereas those with low serum EV-BCMA had a 20-month PFS of 74.9% (95% CI, 39.1–91.5) (*p* = 0.104).

Notably, combining serum EV-IL5RA and EV-BCMA enabled clearer stratification of prognosis. Patients were categorized into three groups: Group 1 (EV-IL5RAlow, EV-BCMAlow), Group 2 ([EV-IL5RAhigh, EV-BCMAlow] or [EV-IL5RAlow, EV-BCMAhigh]), and Group 3 (EV-IL5RAhigh, EV-BCMAhigh). Median PFS and a 20-month PFS for each group were: not reached and 100% for Group 1, 25 months (95% CI, 17-NA) and 67.7% (95% CI, 34.9–86.5) for Group 2, and 14 months (95% CI, 1-NA) and 0% for Group 3 (*p* = 0.001, Figure 3C).

To determine whether serum EV-IL5RA and EV-BCMA serve as independent predictors of PFS, multivariable Cox regression analysis was performed (nine progression events), incorporating age, bone marrow plasma cell percentage, revised International Staging System (R-ISS) [10], and serum soluble BCMA (sBCMA). sBCMA was included in the model based on previous reports suggesting the association with tumor burden and prognosis in MM [26,27,28], to enable comparison with EV-derived biomarkers. Consistent with the previous reports, sBCMA was significantly higher in MM patients (*n* = 26) compared to healthy individuals (*n* = 60) (Figure S4). However, in our cohort of NDMM patients treated with daratumumab-containing regimens, sBCMA levels did not correlate with PFS (Figure S5).

Among all variables included in the model, the combination of serum EV-IL5RA and EV-BCMA remained a statistically significant independent prognostic factor in this small exploratory cohort, although this finding requires validation in larger datasets (HR = 38.49 [95% CI, 1.51–47.79], *p* = 0.015; Table 3), underscoring their potential clinical utility. No significant multicollinearity was observed among the covariates included in the multivariable model (all VIFs < 2).

Together, these findings suggest that the combined assessment of serum EV-IL5RA and EV-BCMA provides meaningful prognostic stratification in NDMM receiving daratumumab-based therapy.

### 3.6. IL5RA and BCMA Are Specifically Elevated in MM Cells

**Figure 4:**
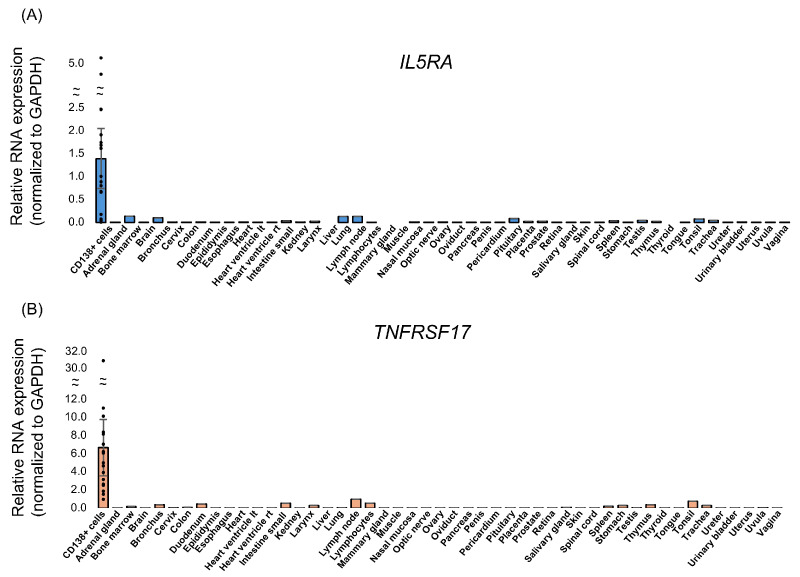
mRNA expression of (**A**) *IL5RA* and (**B**) *TNFRSF17* in CD138-positive bone marrow cells from MM patients compared with 48 normal human tissues using RT-qPCR. Relative expression levels were normalized to GAPDH using the 2^−ΔCT^ method. Error bars represent ±2 × SEM.

To validate their cellular origin, we assessed IL5RA and BCMA expression in CD138+ MM cells using RT-qPCR and compared these levels with those in normal human tissues. Both genes were markedly overexpressed in MM cells. Although low expression was detectable in select lymphoid tissues (e.g., bone marrow, lymph nodes, tonsils), their levels were substantially higher in MM cells, supporting their specificity as MM biomarkers (Figure 4).

## 4. Discussion

In the discovery phase of this study, we comprehensively characterized the proteomic landscape of bone marrow-derived EVs of MM patients and controls. The identification of more than 8800 proteins from the bone marrow EVs, which represents, to our knowledge, the most extensive proteomic dataset of bone marrow EVs reported to date, highlights the molecular complexity of the EV compartment within the myeloma microenvironment. Previous studies have profiled EVs specifically from bone marrow mesenchymal stem cells [29,30,31]. However, our study is distinct in that we analyzed EVs isolated from whole bone marrow aspirate supernatants, which likely include EVs secreted not only by plasma cells but also by stromal and immune cells within the bone marrow microenvironment. In contrast, Harshman et al. conducted LC-MS analysis of bone marrow and serum EVs from two MM patients and reported 607 proteins, including enriched MHC class I in MM-derived EVs [32]. Our study significantly expands on these findings by presenting a comprehensive EV proteome from nine MM patients compared to healthy controls.

From this large-scale dataset, EV-IL5RA and EV-BCMA emerged as promising prognostic biomarkers detectable in serum-derived EVs from NDMM patients. The combined evaluation of these serum EV biomarkers enabled clear stratification of patients into distinct prognostic groups, including a cohort with remarkably poor outcomes. Notably, the combined EV-IL5RA and EV-BCMA signature retained prognostic significance after adjustment for established clinical parameters, suggesting that these EV biomarkers may provide prognostic information that is not fully captured by conventional risk factors. It should be noted that, given the small number of events (*n* = 9) relative to the five covariates included, the multivariable model may be susceptible to overfitting. Furthermore, because the biomarker cut-offs were derived using ROC analysis within this cohort, they lack external validation. Therefore, these prognostic values should be interpreted as exploratory findings. Nevertheless, these findings suggest that serum EV-IL5RA and EV-BCMA are clinically meaningful prognostic biomarkers with strong potential to improve risk stratification in NDMM, particularly in the era of antibody-based frontline therapy.

Notably, this biomarker combination may serve as a powerful tool to identify high-risk patients who might benefit from closer monitoring, early treatment intensification or enrollment in clinical trials. Our findings offer a foundation for further studies aimed at refining patient selection in such clinical trials and underscore the clinical relevance of EV-based biomarker strategies.

Currently, MM diagnosis and risk assessment rely heavily on bone marrow aspiration, which is invasive and occasionally yields insufficient material. Moreover, well-established cytogenetic abnormalities such as del(17p), t(4;14), t(14;16), and gain/amp(1q) are prognostically informative [10,33], but their detection also requires bone marrow samples and may not fully capture disease heterogeneity across different sites of involvement, including bone lesions or extramedullary disease [11,34]. Our results indicate that serum EV-derived IL5RA and BCMA, obtainable via routine blood tests, can serve as less-invasive biomarkers and may reflect a more comprehensive picture of disease biology.

BCMA is a well-established therapeutic target in MM and is highly expressed on plasma cells [35]. Therapeutic approaches targeting BCMA include antibody-drug conjugates [36], CAR-T cell therapy [37,38], and bispecific antibodies [39,40]. While the soluble form of BCMA, generated through γ-secretase cleavage, is known to be elevated in MM and associated with inferior outcomes [26,27], our study is the first to report its enrichment in EVs from MM patients. This may provide a novel perspective on BCMA trafficking and its extracellular roles. Because sBCMA has been reported as a biomarker in MM, we also examined the relationship between sBCMA and EV-BCMA. No clear correlation was observed between these two measurements (Figure S6). sBCMA is primarily generated through the shedding of surface BCMA by γ-secretase, whereas EV-BCMA is released via the active cellular secretion of EVs. Therefore, this lack of correlation suggests that these two forms of circulating BCMA are produced and cleared through different mechanisms, reflecting distinct biological processes.

The role of IL5RA in MM pathogenesis has not been fully elucidated. IL5RA, together with IL5βc, forms the receptor for interleukin-5 (IL-5). Upon IL-5 binding, the receptor complex activates intracellular signaling via JAK2 and STAT5 pathways [41,42,43], leading to upregulation of anti-apoptotic genes (e.g., BCL2L1, BCL-2), cell proliferation drivers (e.g., MYC, PIM1), and cell cycle regulators (e.g., CCND1) [44]. Based on these known mechanisms, we hypothesize that IL5RA may promote MM cell survival and proliferation, thereby contributing to disease progression and poor prognosis. However, the current study did not include functional experiments to directly validate these mechanisms; therefore, the proposed interpretation remains speculative. Further mechanistic studies will be required to clarify the biological role of IL5RA in MM pathogenesis.

Additionally, IL5RA expression was substantially elevated in MM cells compared to normal tissues, suggesting that IL5RA could be a promising therapeutic target worthy of further investigation.

The main limitations of our study include the small sample size and a relatively short follow-up duration. Moreover, the limited number of events may increase the risk of overfitting. Therefore, the results should be validated in a larger, independent cohort. The median follow-up period of 20 months may be insufficient in the context of novel therapies, in which median PFS often exceeds five years [6,7]; therefore, longer follow-up will be required to confirm the long-term prognostic value of these biomarkers. Moreover, the daratumumab-containing quadruplet regimen for transplant-eligible patients was only recently approved [45], and such patients were not included in our cohort. Therefore, caution is required when applying our findings to transplant-eligible populations.

Nevertheless, the ability of serum EV-IL5RA and EV-BCMA and IL5RA to identify patients with early relapse supports their potential value in real-world clinical decision-making.

## 5. Conclusions

We identified novel EV-derived biomarkers, IL5RA and BCMA, that are detectable through non-invasive blood tests in patients with newly diagnosed MM. The combined assessment of serum EV-IL5RA and EV-BCMA enabled meaningful prognostic stratification, allowing identification of patients with markedly inferior outcomes. This suggests potential utility in guiding treatment decisions. Future studies should aim to validate these findings in larger cohorts and investigate the utility of longitudinal EV biomarker monitoring in predicting treatment response and early relapse.

## Figures and Tables

**Table 1:** List of biomarker candidate proteins identified through label-free quantification of bone marrow EVs selected from proteins exclusively detected in bone marrow EVs of MM patients.

Uniprot ID	Protein Name	Gene Name
Q6UXG2	Endosome/lysosome-associated apoptosis and autophagy regulator 1	ELAPOR1
Q01344	Interleukin-5 receptor subunit alpha	IL5RA
Q9P2E3	NFX1-type zinc finger-containing protein 1	ZNFX1
P0C7P3	Protein SLFN14	SLFN14
Q8TDP1	Ribonuclease H2 subunit C	RNASEH2C
O00220	Tumor necrosis factor receptor superfamily member 10A	TNFRSF10A
Q02223	Tumor necrosis factor receptor superfamily member 17	TNFRSF17
Q6RFH5	WD repeat-containing protein 74	WDR74

Proteins meeting the criteria of unique peptides ≥ 2 and a valid value ≥ 50% were selected.

**Table 2:** List of biomarker candidate proteins identified through label-free quantification of bone marrow EVs selected from proteins significantly upregulated in EVs of MM patients.

Uniprot ID	Protein Name	Gene Name	FC ^(a)^	*p*-Value ^(b)^
Q86X29	Lipolysis-stimulated lipoprotein receptor	LSR	21.5	0.040
Q8WVR3	Trafficking protein particle complex subunit 14	TRAPPC14	14.9	0.045
Q6ICL3	Transport and Golgi organization protein 2 homolog	TANGO2	11.8	0.020
P15907	Beta-galactoside alpha-2,6-sialyltransferase 1	ST6GAL1	11.4	0.014
Q15035	Translocating chain-associated membrane protein 2	TRAM2	10.9	0.048
Q8WU39	Marginal zone B- and B1-cell-specific protein	MZB1	10.5	0.039

Proteins meeting the criteria of unique peptides ≥ 2, a valid value ≥ 50%, and a fold change ≥ 10 were selected. ^(a)^ The proportion of the averaged relative bone marrow EV protein abundances of bone marrow in MM to that of the control. ^(b)^ *p*-values were calculated using two-tailed Welch’s *t*-test for differential analysis of MM and control.

**Table 3:** Multivariable analysis of factors associated with progression-free survival.

Factor	Hazard Ratio	95% CI	*p* Value
Age	1.00	(0.91–1.10)	0.96
Serum EV-IL5RA and EV-BCMA	8.49	(1.51–47.79)	0.015
Serum soluble BCMA	0.83	(0.13–5.22)	0.84
Bone marrow plasma cells (%)	1.00	(0.96–1.03)	0.82
R-ISS	15.10	(0.84–272.50)	0.066

R-ISS: revised international staging system, including serum beta-2 microglobulin, serum albumin, serum lactate dehydrogenase, and chromosomal abnormalities.

## Data Availability

The LC-MS raw data were deposited in the Japan Proteome Standard Repository/Database (jPOST), JPST003913.

## Supplementary Materials

The following supporting information can be downloaded at: https://www.mdpi.com/article/10.3390/cancers18071116/s1, Figure S1: Full-length Western blot images corresponding to Figure 1B, including molecular weight markers; Figure S2: Comparison of M-protein levels according to (a) serum EV-IL5RA status, (b) serum EV-BCMA status, and (c) their combination. Differences between groups were evaluated using the Mann–Whitney U test; Figure S3: ROC curve for each serum EV protein and soluble BCMA as a predictor of progression-free survival. (a) serum EV-IL5RA, (b) serum EV-BCMA, (c) serum soluble BCMA; Figure S4: Serum soluble BCMA concentrations in healthy control vs. MM patients; Figure S5: Progression-free survival of NDMM patients according to serum soluble BCMA level; Figure S6: Correlation between serum EV-BCMA and soluble BCMA (sBCMA) assessed by Spearman’s rank correlation analysis; Table S1A: Characteristics of MM patients included in the bone marrow EV analysis title; Table S1B: Characteristics of MM patients treated with daratumumab-containing regimens included in the serum EV analysis; Table S2A: Characteristics of lymphoma patients included in the bone marrow EV analysis as controls; Table S2B: Characteristics of healthy individuals included in the serum EV analysis as controls; Table S3: Multiple reaction monitoring parameters; Table S4: The list of 8839 identified EV-proteins: Table S5A: List of EV proteins exclusively detected in MM bone marrow; Table S5B: List of EV proteins exclusively detected in control bone marrow; Table S6A: List of significantly up-regulated EV proteins in MM bone marrow compared to control bone marrow ordered by descending FC; Table S6B: List of significantly down-regulated EV proteins in MM bone marrow compared to control bone marrow ordered by ascending FC; Table S7A: Enriched GO terms of biological process; Table S7B: Enriched GO terms of cellular component; Table S7C: Enriched GO terms of molecular function.

## Abbreviations

The following abbreviations are used in this manuscript:

BCMA	B-cell maturation antigen
EV	Extracellular vesicles
IL5RA	Interleukin-5 receptor alpha
MM	Multiple myeloma
MRM	Multiple reaction monitoring
NDMM	Newly diagnosed multiple myeloma
OS	Overall survival
PFS	Progression-free survival
R-ISS	Revised international staging system
sBCMA	Soluble B-cell maturation antigen
