# Supplementary material for: Extracellular Vesicle IL5RA and BCMA in Serum Enable Non-Invasive Risk Stratification of Multiple Myeloma
Source: Cancers (Basel). 2026 Mar 30;18(7):1116. doi: 10.3390/cancers18071116 (PMC13072286; doi:10.3390/cancers18071116)
Supplement: Supplementary file 1 [file cancers-18-01116-s001.zip › Supplementary figures_R1.pdf]

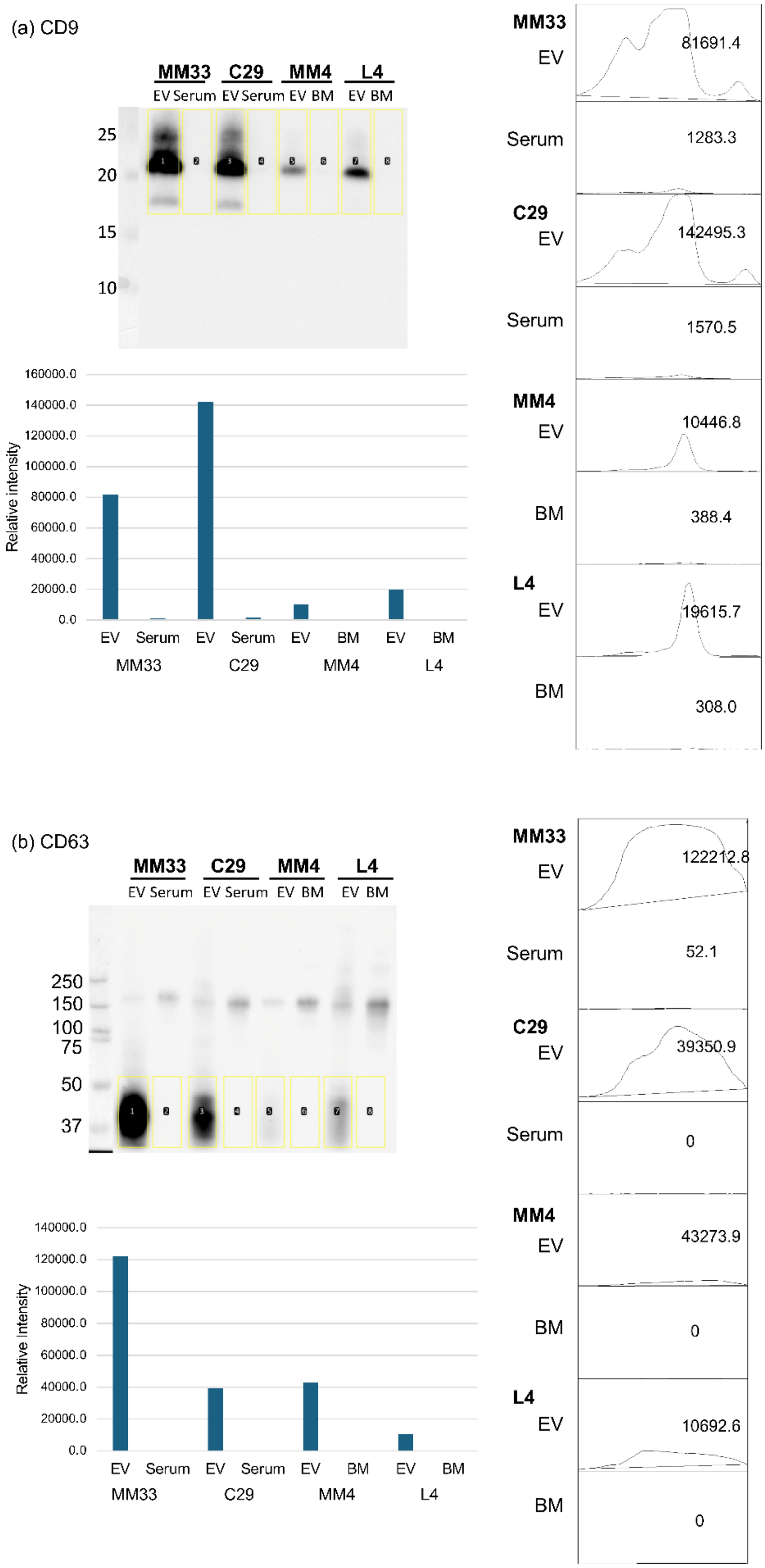
(a) CD9
MM33 C29 MM4 L4
EV Serum EV Serum EV BM EV BM
25
20
15
10
Relative intensity
160000.0
140000.0
120000.0
100000.0
80000.0
60000.0
40000.0
20000.0
0.0
EV Serum EV Serum EV BM EV BM
MM33 C29 MM4 L4
MM33 EV 81691.4
Serum 1283.3
C29 EV 142495.3
Serum 1570.5
MM4 EV 10446.8
BM 388.4
L4 EV 19615.7
BM 308.0
(b) CD63
MM33 C29 MM4 L4
EV Serum EV Serum EV BM EV BM
250
150
100
75
50
37
Relative Intensity
140000.0
120000.0
100000.0
80000.0
60000.0
40000.0
20000.0
0.0
EV Serum EV Serum EV BM EV BM
MM33 C29 MM4 L4
MM33 EV 122212.8
Serum 52.1
C29 EV 39350.9
Serum 0
MM4 EV 43273.9
BM 0
L4 EV 10692.6
BM 0

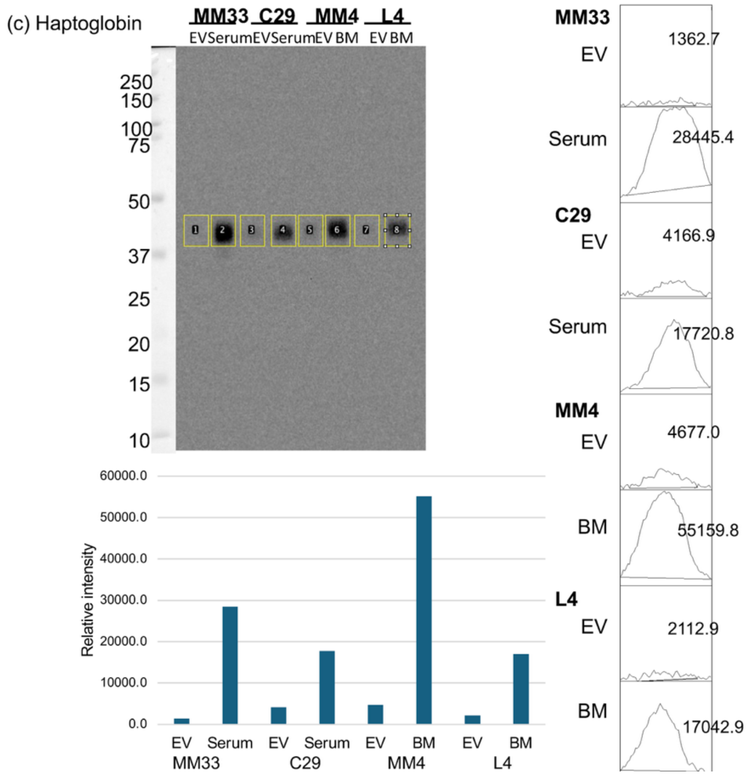

Figure S1. Full-length Western blot images corresponding to Figure 1B, including molecular weight markers, and corresponding densitometry quantification performed using image J.

a)

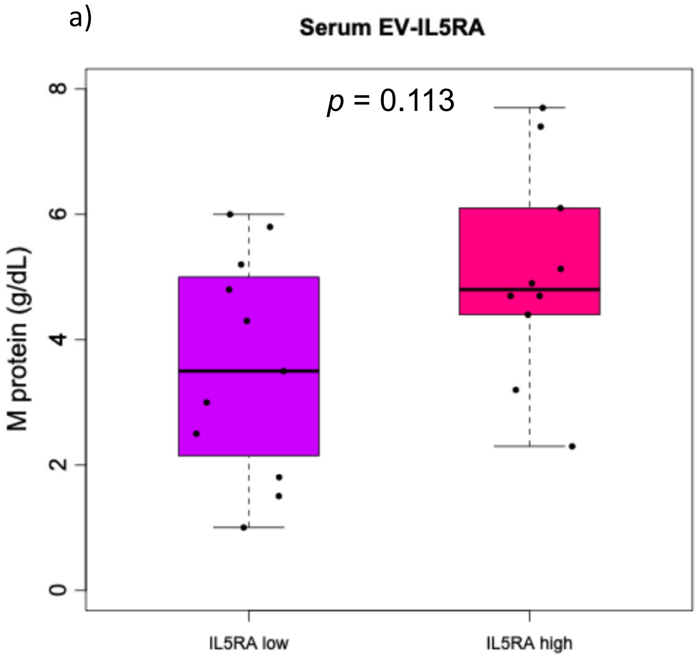

b)

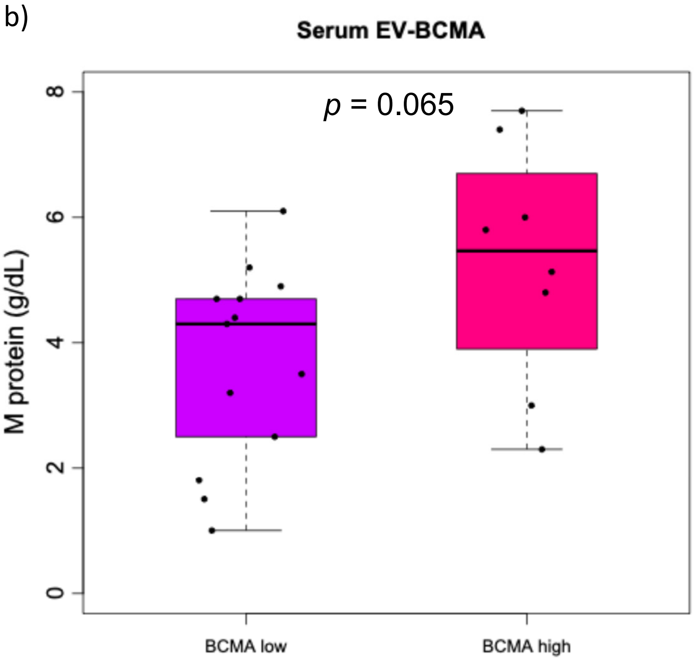

c)

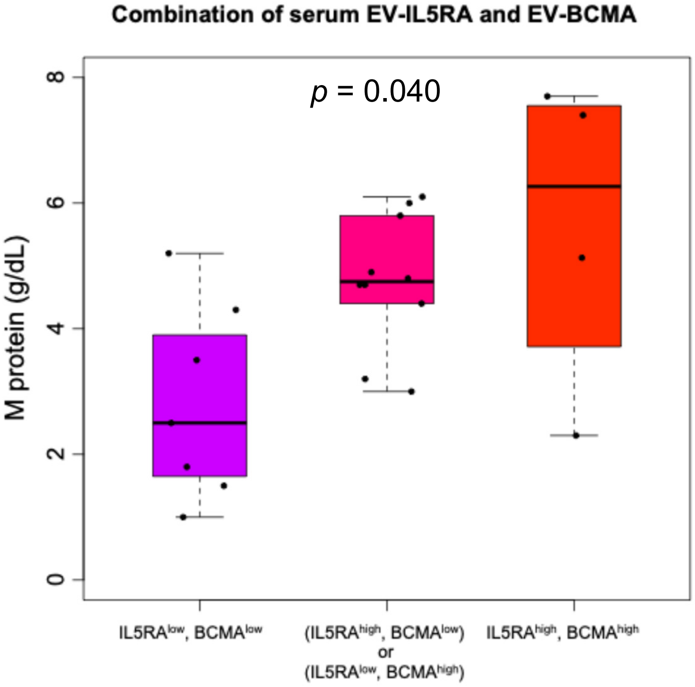

Figure S2. Comparison of M-protein levels according to (a) serum EV-IL5RA status, (b) serum EV-BCMA status, and (c) their combination. Differences between two groups were assessed using the Mann-Whitney U test, and comparisons among three groups were performed using the Kruskal-Wallis test.

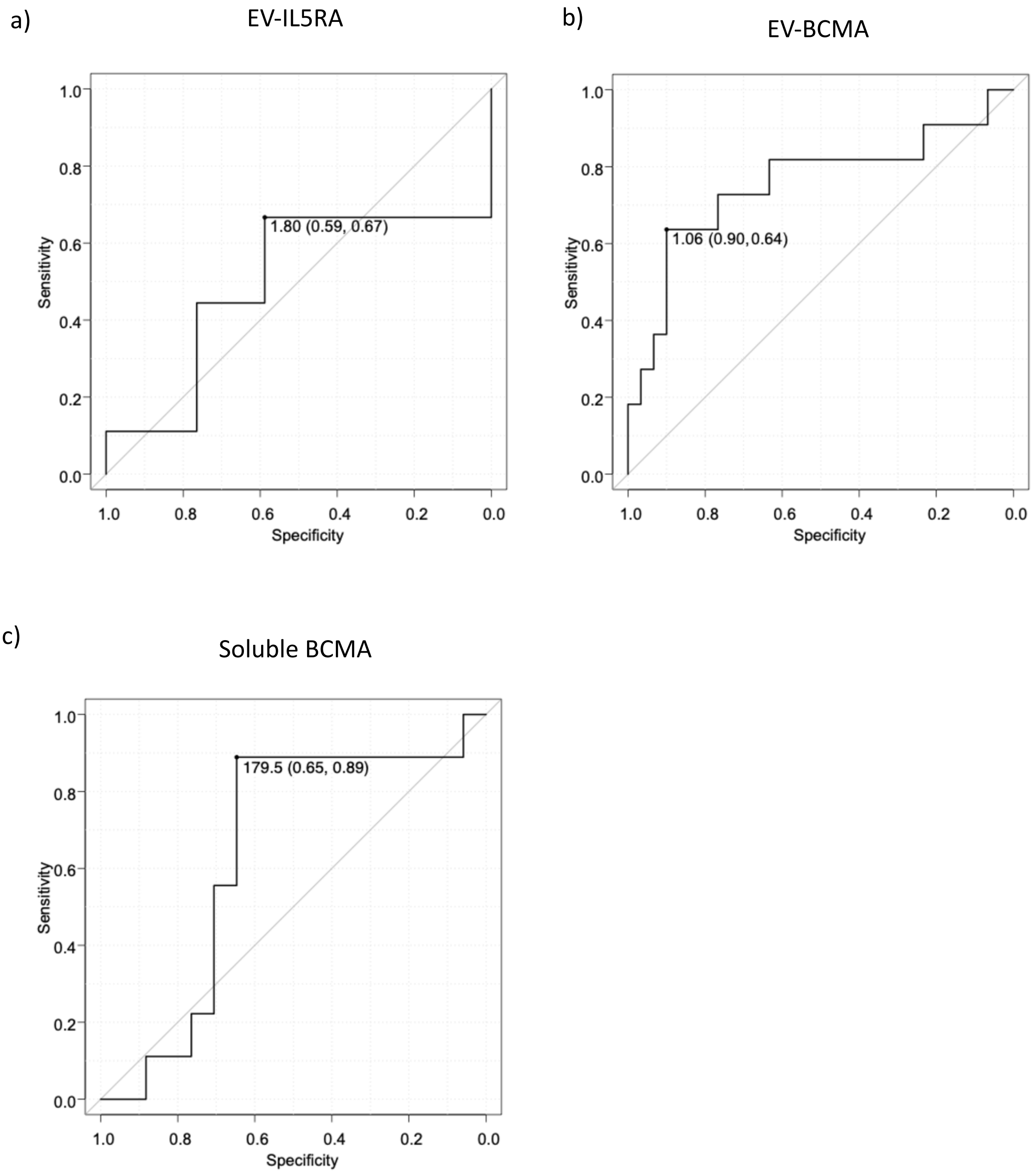

Figure S3. ROC curve for each serum EV protein and soluble BCMA as a predictor of progression-free survival. a) serum EV-IL5RA, b) serum EV-BCMA, c) serum soluble BCMA

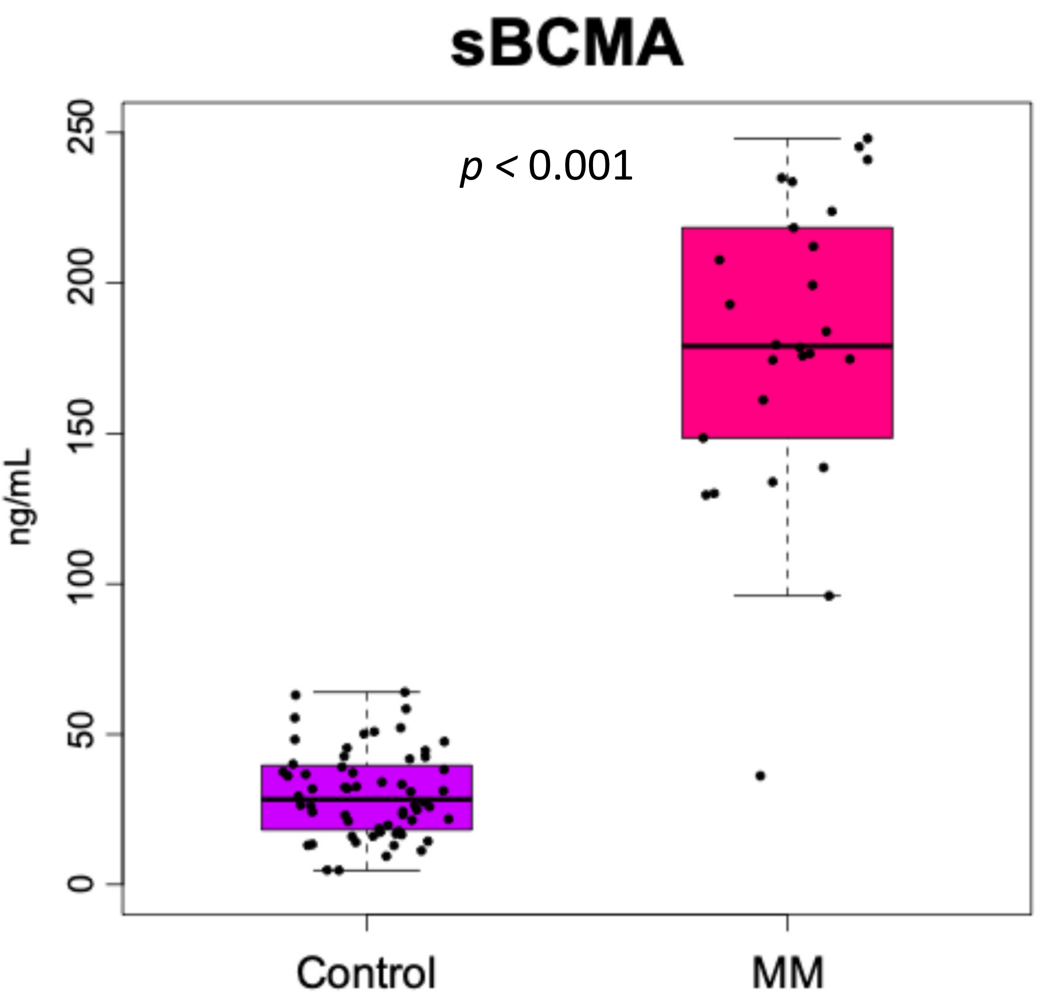

Figure S4. Serum soluble BCMA concentrations in healthy control vs. MM patients.

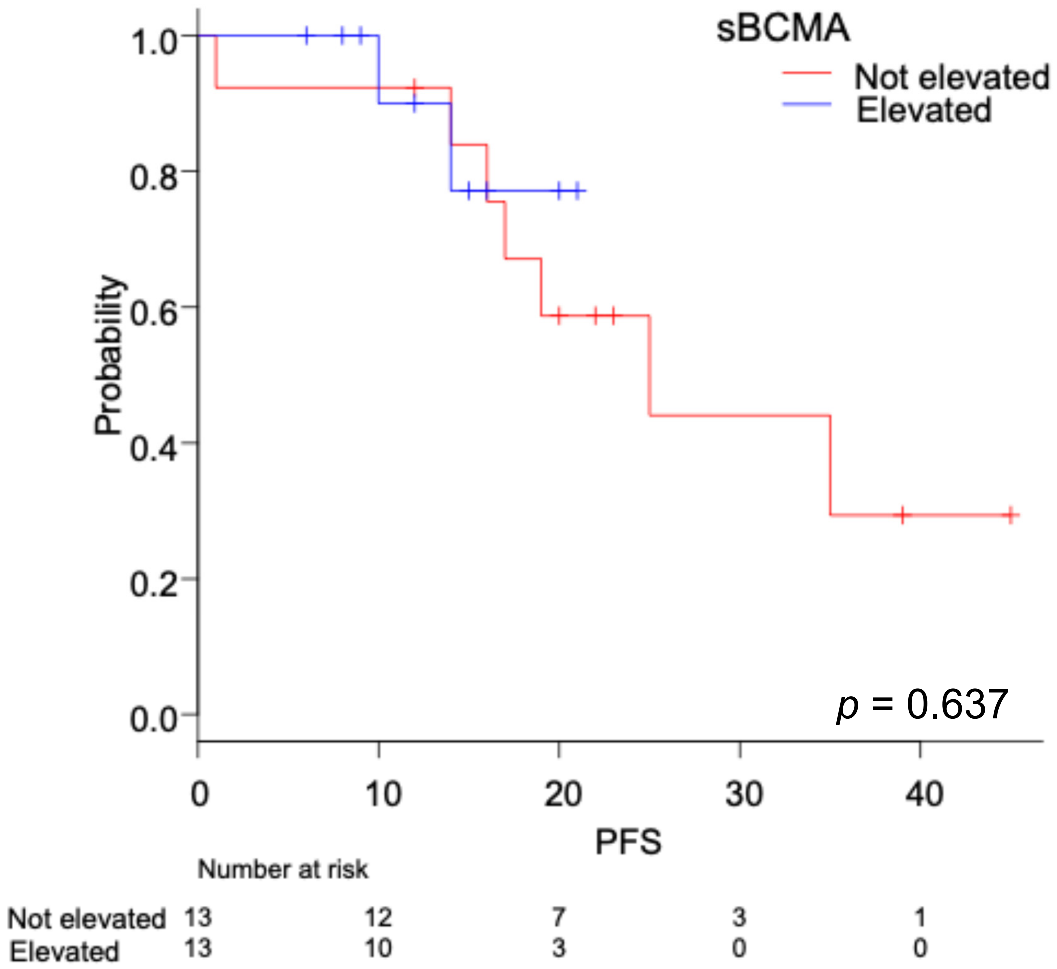

Figure S5. Progression-free survival of NDMM patients according to serum soluble BCMA level

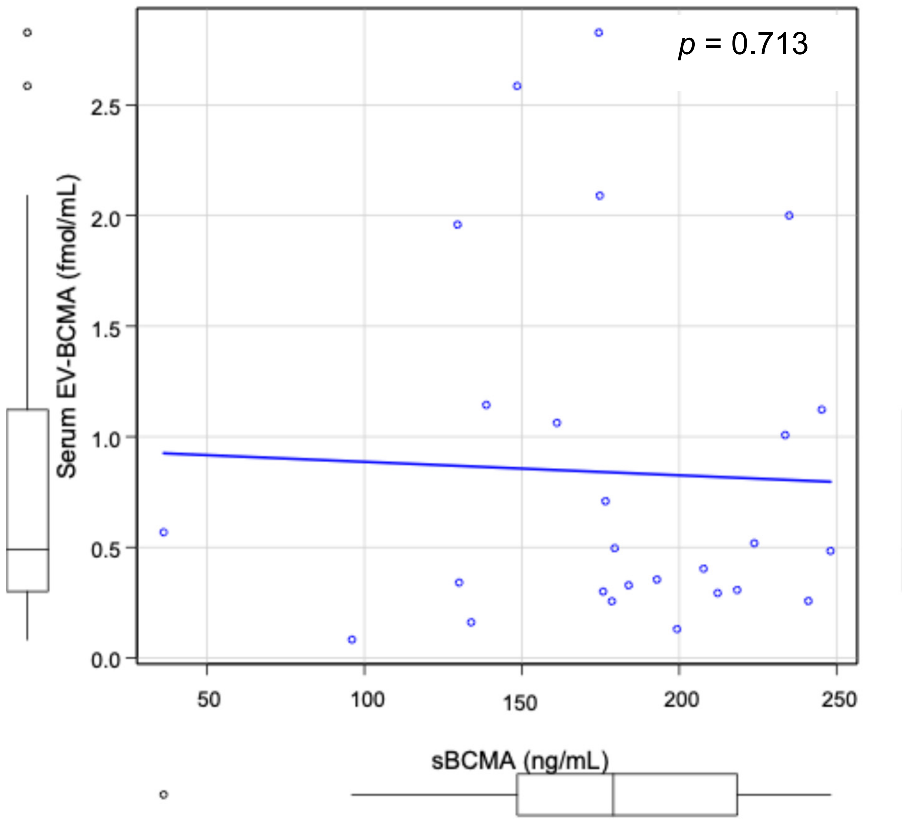

Figure S6. Correlation between serum EV-BCMA and soluble BCMA (sBCMA) assessed by Spearman's rank correlation analysis.
